# Preparation and Performance Study of Low Drive Voltage, Wide-Temperature Stable PDLC Films

**DOI:** 10.3390/molecules31091402

**Published:** 2026-04-23

**Authors:** Haokai Wang, Wanghan Sheng, Shikang Zhang, Guanqiao Wang, Yanjun Zhang

**Affiliations:** School of Science, Hebei University of Technology, Tianjin 300401, China; 18931890722@163.com (H.W.); 13739174400@163.com (W.S.); 15169123793@163.com (S.Z.); 18605356845@163.com (G.W.)

**Keywords:** polymer-dispersed liquid crystal, low driving voltage, electro-optical properties, high-temperature stability

## Abstract

Traditional polymer-dispersed liquid crystal (PDLC) faces limitations in smart dimming applications due to high driving voltage and poor high-temperature stability. In this study, a high-birefringence liquid crystal (QYPDLC-901) was used to prepare PDLC films with liquid crystal contents ranging from 72 wt% to 80 wt%, achieved through synergistic regulation of a low-functional acrylic polymer system and a low-intensity curing process. The effects of liquid crystal content, cell gap, and temperature on electro-optical properties were systematically investigated. Optimal performance was obtained at a liquid crystal content of 77 wt%, with a low threshold voltage of 2.9 V, saturation voltage of 7 V, fast response (rise time 4.2 ms, decay time 47 ms), and a favorable balance between high on-state and low off-state transmittance. Microstructural analysis revealed that the superior performance results from uniform droplet dispersion and low interfacial energy. Furthermore, the PDLC exhibited excellent switching stability from 23 °C to 90 °C, maintaining a maximum transmittance of 93% at 90 °C, with increases of only 0.4 V in threshold voltage and 0.1 V in saturation voltage. This study provides an experimental basis for designing smart dimming devices suitable for low-voltage driving and extreme environments.

## 1. Introduction

Polymer-dispersed liquid crystal (PDLC) is a composite photoelectric material constructed by uniformly dispersing liquid crystal microdroplets in a solid polymer matrix, combining the photoelectric sensitivity of liquid crystals with the excellent film-forming properties of polymers [1]. The core electro-optic modulation mechanism of this material does not rely on polarizers but is based on the refractive index matching effect induced by an external electric field [2]. In the off-state (no external electric field), the liquid crystal molecules within the droplets adopt a random orientation. This random alignment creates a mismatch between the effective refractive index of the liquid crystal and that of the polymer matrix, resulting in strong broadband light scattering and a macroscopically opaque appearance. When an alternating electric field is applied (on-state), the liquid crystal molecules align with the field direction. Under this condition, the ordinary refractive index of the liquid crystal approaches that of the polymer matrix, interfacial scattering is greatly reduced, and the film rapidly becomes highly transparent [3]. Leveraging its fast and reversible dynamic light scattering characteristics, PDLC materials have demonstrated irreplaceable application value and broad development prospects in cutting-edge fields such as smart dimming windows [4,5], variable light attenuators [6,7], photoelectric sensors [8,9], and holographic display devices [10,11].

In recent years, to break through the performance bottlenecks of traditional PDLC in practical applications, researchers have conducted extensive cutting-edge explorations around core demands such as reducing driving voltage and wide-temperature-range stability. From the perspective of polymer matrix engineering, the introduction of low-functional acrylic monomers with heteroatom-terminated groups or hydrogen-bonding moieties has been demonstrated to significantly reduce threshold and saturation voltages by weakening interfacial anchoring energy while maintaining adequate phase separation morphology [12,13]. For instance, Mhatre et al. reported that fluorinated acrylate systems can lower the threshold voltage by nearly 40% and the saturation voltage by 30% in 20 μm films, achieving a contrast ratio exceeding 100 [3]. Similarly, epoxy–mercaptan-based phase-separated composites have been shown to yield high contrast ratios at remarkably low driving fields owing to the formation of uniform microdroplet morphologies and reduced interfacial tension [14]. In parallel, nanoparticle doping strategies offer an alternative pathway for voltage reduction and thermal stabilization. The incorporation of ZnO quantum dots or gold nanoparticles has been found to decrease the switching voltage by locally enhancing the effective electric field and modulating the dielectric response of the liquid crystal domains [15,16], while multiwalled carbon nanotubes and MgO nanoparticles accelerate the dynamic response and broaden the operational frequency range [17,18]. Concerning wide-temperature stability, the choice of liquid crystal components with high clearing points and the optimization of curing protocols near the nematic–isotropic transition temperature is critical [19,20]. Xu et al. demonstrated that the use of fluorinated high-birefringence liquid crystals can extend the phase transition temperature above 82 °C, enabling the film to maintain a low driving voltage (<20 V) and high contrast ratio (>70) over the 0–60 °C range [21]. Additionally, crosslinking monomer selection and curing progress profoundly influence the temperature-dependent electro-optic behavior; networks with higher crosslink density tend to preserve structural integrity and scattering efficiency at elevated temperatures, albeit at the expense of slightly increased driving voltage [22,23].

However, despite the significant achievements of the aforementioned modification strategies in enhancing specific performance of PDLC or enabling multifunctionality, current optimization schemes often struggle to balance low driving voltage and wide-temperature-range stability, limiting the application of PDLC in harsh outdoor scenarios (such as smart vehicle glass, building facades, etc.) [1,24,25]. From the perspective of phase separation thermodynamics, conventional approaches for achieving low driving voltages typically rely on reducing the crosslink density of the polymer network or incorporating highly flexible chain segments. These strategies aim to weaken the interfacial anchoring exerted by the polymer matrix on the liquid crystal molecules [3]. However, such molecular designs inevitably sacrifice the rigidity of the matrix, leading to a substantial decline in the thermal and mechanical stability of the composite system [22]. In actual outdoor high-temperature service environments (such as ≥80 °C), low-crosslinked or highly flexible polymer skeletons are prone to thermal softening, which further triggers irreversible instability of the internal microphase separation structure, specifically manifested as the disordered merging of liquid crystal microdroplets and the sharp dissipation of interfacial anchoring energy [19]. On a macro scale, this not only leads to a sharp reduction in the light scattering cross-section of PDLC films in the off-state, but also induces a severe shift in the threshold voltage, causing it to completely lose its reliable photoelectric modulation capability over a wide temperature range [25,26]. Therefore, reconciling low driving voltage with high-temperature structural stability remains a significant challenge in the development of advanced PDLC materials. Constructing a composite system that maintains both excellent electro-optical response and microstructural integrity across a wide temperature range represents a key technical bottleneck for this field [7,24].

To address the aforementioned challenges, this paper employs molecular design of a low-functional acrylic system, using epoxy acrylate (CN131) as the polymer backbone, blending monofunctional active diluents (OPPEA, IBOA) and trifunctional crosslinking agents (TMPTA), and incorporating high birefringence liquid crystals (QYPDLC-901), aiming to prepare a PDLC film that combines low driving voltage and wide-temperature stability. A systematic study was conducted on the influence of liquid crystal content and cell thickness on the microstructure and electro-optical properties of PDLC, and the working characteristics of the film under a wide temperature range were further analyzed, aiming to provide an experimental basis for the design of smart dimming devices suitable for harsh environments such as automotive and outdoor applications.

## 2. Results and Discussion

### 2.1. Effect of LC Content on the Electro-Optical Performance of PDLC

**Figure 1:**
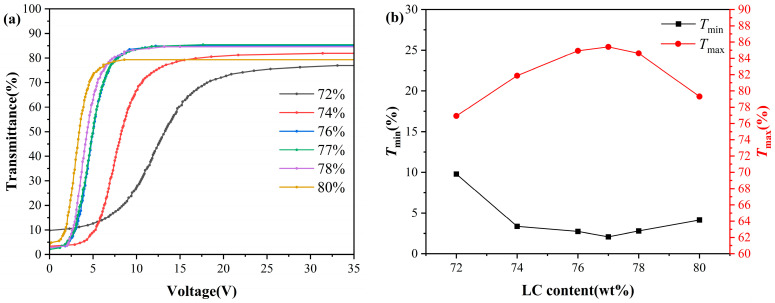

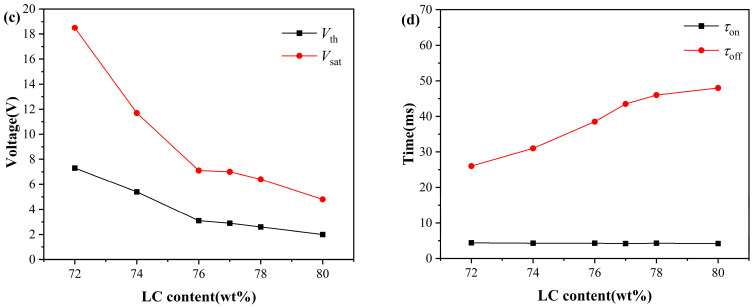
Electro-optical properties of PDLCs with different liquid crystal contents: (**a**) voltage-transmittance curve; (**b**) maximum and minimum transmittance; (**c**) driving voltage; (**d**) response time.

To systematically investigate the effect of liquid crystal content on the electro-optical properties of PDLC films, this study prepared PDLC films with different liquid crystal contents and systematically characterized their electro-optical properties and microstructure. Figure 1 shows the variation of transmittance, driving voltage, and response time of PDLC films with a cell gap of 4 μm as a function of liquid crystal content ranging from 72 wt% to 80 wt%.

As shown in Figure 1b, with the liquid crystal content increasing from 72 wt% to 77 wt%, the maximum transmittance ($T_{max}$) of PDLC continuously rises, while the minimum transmittance ($T_{min}$) continuously decreases; after the liquid crystal content exceeds 77 wt%, $T_{max}$ begins to decline, and $T_{min}$ recovers, achieving the optimal match in transmittance at 77 wt%, with contrast reaching its peak. Figure 1c shows that both the threshold voltage ($V_{th}$) and saturation voltage ($V_{sat}$) decrease as the liquid crystal content increases; Figure 1d indicates that the rise time ($\tau_{on}$) remains basically unchanged, while the decay time ($\tau_{off}$) gradually extends. Considering the above results, when the liquid crystal quality fraction is 77%, PDLC achieves the optimal ratio with the highest contrast, lower driving voltage, and faster response time. At this point, $V_{th}$ is as low as 2.9 V, $V_{sat}$ is only 7 V, $\tau_{on}$ is 4.2 ms, and $\tau_{off}$ is 47 ms.

**Figure 2:**
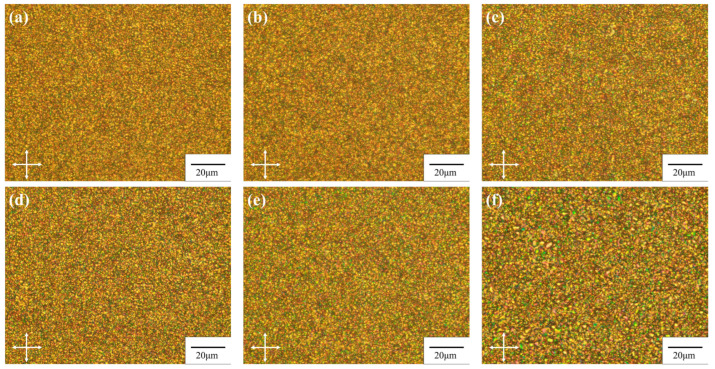
POM images of PDLC films with different liquid crystal contents: (**a**) 72 wt%, (**b**) 74 wt%, (**c**) 76 wt%, (**d**) 77 wt%, (**e**) 78 wt%, (**f**) 80 wt%.

The microstructure of PDLC is determined by phase separation dynamics and regulated by factors such as system viscosity, curing rate, and component diffusion, which are closely related to liquid crystal content, pre-polymer ratio, and curing process [20,27,28]. Among them, the liquid crystal content is a key parameter affecting the polymer network structure, as it significantly alters the system viscosity and polymerization reaction kinetics [14,29]. Figure 2 presents the polarizing optical microscope (POM) images of PDLC films with a cell gap of 4 μm, prepared with different liquid crystal contents. When the liquid crystal content increases from 72 wt% to 77 wt%, the size of the liquid crystal droplets shows a gradual increasing trend. When the liquid crystal content exceeds 77 wt%, a further increase in the apparent size of the liquid crystal domains is observed in the POM images. This observation may be attributed to the further growth and coalescence of neighboring microdroplets, suggesting a possible transition of the microstructure from an isolated droplet morphology toward a more interconnected network. Consequently, the continuity of the polymer network is likely reduced.

As shown in Figure 1b, the changes in the microstructure correlate with the variations in device transmittance. When the liquid crystal content increases from 72 wt% to 77 wt%, $T_{min}$ continues to decrease. This may be attributed to the observed increase in droplet size, which likely enhances the light scattering cross-section within this range, thereby improving the off-state light-shielding ability of the film. Meanwhile, $T_{max}$ gradually increases over the same range, which is speculated to result from more thorough phase separation, leading to higher purity of the liquid crystal phase and improved orientational order under an electric field [30]. When the liquid crystal content is further increased beyond 77 wt%, $T_{min}$ rebounds while $T_{max}$ declines. This trend is tentatively attributed to the following factors. On the one hand, the further increase in droplet size observed in the polarizing optical microscope images—suggesting possible droplet coalescence and a consequent reduction in polymer network continuity—may hinder the uniform orientation of liquid crystal domains under an electric field, thereby increasing residual scattering [31]. On the other hand, an excessively high liquid crystal content may disturb the kinetic equilibrium of phase separation, leaving a small amount of uncured prepolymer within the liquid crystal phase, which adversely affects refractive index matching between the liquid crystal and the polymer matrix [32]. Collectively, these factors are considered responsible for the transition in transmittance behavior observed within this composition range.

The drive voltage of PDLC is inversely proportional to the radius of the liquid crystal microdroplets (R), as shown in the following formula [33]:

(1) $$\begin{array}{c} V_{\mathrm{th}}\approx \frac{d}{R}{\left[\frac{K\left(\omega^{2}-1\right)}{\varepsilon_{0}\Delta \varepsilon }\right]}^{1/2} \end{array}$$

(2) $$\begin{array}{c} V_{\mathrm{sat}}\approx \frac{d}{R}{\left(\omega^{2}-1\right)}^{1/2}\frac{4\pi K}{\Delta \varepsilon } \end{array}$$

where *d* is the membrane thickness, *K* is the liquid crystal elastic constant, *ω* is the droplet aspect ratio, $\varepsilon_{0}$ is the vacuum permittivity, and Δε is the liquid crystal dielectric anisotropy. The response time is described by the following formula [33]:

(3) $$\begin{array}{c} \tau_{\mathrm{on}}\approx \frac{\gamma }{\Delta \varepsilon V^{2}-\frac{K\left(l^{2}-1\right)}{R^{2}}} \end{array}$$

(4) $$\begin{array}{c} \tau_{\mathrm{off}}\approx \frac{R^{2}\gamma }{K\left(l^{2}-1\right)} \end{array}$$

where *V* is the applied electric field strength, *K*, l, γ, and ∆ε represent the elastic constants, shape anisotropy, rotation viscosity constant, and dielectric anisotropy of the liquid crystal, respectively. Equations (1)–(4) indicate that an increase in droplet radius can reduce $V_{th}$, $V_{sat}$, and $\tau_{on}$, but will prolong $\tau_{off}$, which is attributed to the weakening anchoring force of the polymer matrix on the liquid crystal molecules as the droplet size increases [34].

The experimental results of the driving voltage in this study are consistent with the theoretical predictions of Equations (1) and (2), where $V_{th}$, and $V_{sat}\;$decrease as the droplet size increases. In terms of response time, $\tau_{off}$ extends with increasing droplet size, consistent with Equation (4); however, $\tau_{on}$ does not decrease with increasing droplet size but remains largely unchanged. This deviation can be explained as follows: in high liquid crystal content systems, the polymer exerts a weaker anchoring force on the liquid crystal molecules, requiring only a lower voltage to drive the liquid crystal molecules to deflect. Under the dominance of the applied electric field, the liquid crystal molecules rapidly achieve ordered alignment, making the effect of droplet size on $\tau_{on}$ no longer significant [35].

**Figure 3:**
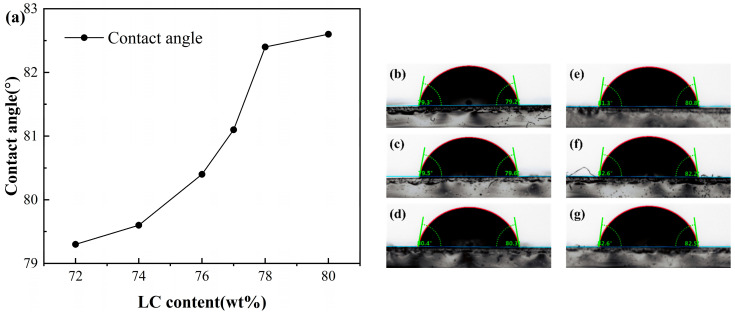
(**a**) Variation of the liquid crystal contact angle of PDLC polymer films with different liquid crystal contents. Contact angle measurements for PDLC with different liquid crystal contents: (**b**) 72 wt%, (**c**) 74 wt%, (**d**) 76 wt%, (**e**) 77 wt%, (**f**) 78 wt%, (**g**) 80 wt%.

To further reveal the effect of liquid crystal content on the surface energy of the polymer matrix, this study employs the static contact angle measurement method. As shown in Figure 3, with the increase in liquid crystal content, the contact angle of deionized water on the polymer/liquid crystal surface gradually rises from 79.3° to 82.6°. According to the Young–Dupré equation [36]:

(5) $$\begin{array}{c} \cos\theta =\frac{\gamma_{SL}-\gamma_{L}}{\gamma_{L}} \end{array}$$

where *θ* is the contact angle $\gamma_{SL}$ is the solid/liquid interfacial free energy, and $\gamma_{L}$ is the liquid surface free energy. A larger contact angle corresponds to a lower surface energy of the polymer matrix. The contact angle results therefore indicate a progressive decrease in interfacial energy with increasing liquid crystal content, reflecting improved compatibility between the two phases.

A direct consequence of reduced interfacial energy is the anticipated weakening of the polymer–liquid crystal interfacial anchoring strength. This interpretation is further supported by the dynamic electro-optical response. The decay time serves as a key parameter characterizing the interaction between the liquid crystal and the polymer matrix. Upon removal of the applied voltage, liquid crystal molecules must overcome interfacial resistance to return to their initial random orientation; the anchoring force exerted by the polymer matrix assists in driving this relaxation process [37]. The observed increase in $\tau_{off}$ with higher liquid crystal content is fully consistent with the proposed reduction in anchoring strength: as the anchoring force weakens, the driving force for relaxation diminishes, thereby prolonging the time required for the liquid crystal molecules to recover their random orientation state.

It is worth noting that all PDLC samples in this study exhibited contact angles close to 90°, confirming their intrinsically low interfacial energy characteristics. This feature also accounts for the lower driving voltages observed in this system.

### 2.2. Cell Gap on the Electro-Optical Performance of PDLC

**Figure 4:**
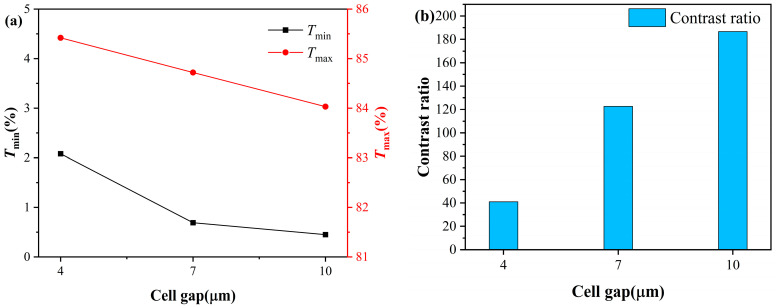
(**a**) Transmittance and (**b**) contrast of PDLCs with different cell thicknesses.

After determining the optimal liquid crystal content to be 77 wt%, this study further investigated the effect of cell gap on the electro-optical performance of PDLC. The transmittance and contrast test results of samples with different cell thicknesses are shown in Figure 4. As the cell thickness increases, both $T_{max}$ and $T_{min}$ continuously decrease, which is due to the increased optical path length with cell gap, leading to an overall enhancement in scattering loss within the film. Notably, the contrast continuously increases with increasing cell thickness, as the decrease in $T_{min}$ is significantly greater than that in $T_{max}$, and the improvement in contrast due to enhanced off-state scattering dominates.

**Figure 5:**
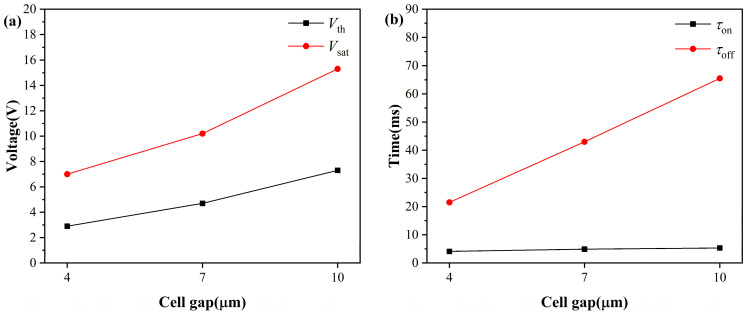
(**a**) Driving voltage, (**b**) response time under different liquid crystal cell thicknesses.

As shown in Figure 5, with the continuous increase in the cell thickness, both the $V_{th}$ and $V_{sat}$ of PDLC exhibit a sustained upward trend. This is because the increase in cell thickness extends the electric field action distance between the ITO electrodes. To achieve effective switching of the liquid crystal molecules from a random orientation state to an electric field-oriented state, a higher voltage needs to be applied to provide sufficient electric field driving torque.

Meanwhile, the decay time of PDLC films shows a continuous extension trend with the increase in cell thickness. After removing the external electric field, the liquid crystal molecules need to recover from the electric field-induced ordered orientation state to the random disordered state under the interfacial anchoring effect. Increasing the cell thickness extends the distance over which the liquid crystal molecules must reorient after the electric field is removed. This prolongs the influence of viscous resistance during relaxation. Moreover, the interfacial anchoring exerted by the polymer network tends to be weaker in thicker films. Both factors contribute to a slower orientational recovery process, resulting in the observed increase in decay time with cell thickness [38]. However, the rise time, dominated by the external electric field, shows little variation with cell thickness.

When PDLC is in the on-state, the orientation of liquid crystal molecules is parallel to the electric field direction, and the liquid crystal droplets behave as uniaxial anisotropic crystals. When light is incident at any angle, the refractive index of the liquid crystal for the transmitted light is between the ordinary light refractive index and the extraordinary light refractive index. At this time, the effective refractive index $n_{eff}$ of the liquid crystal droplets can be expressed as [39]:

(6) $$\begin{array}{c} \frac{1}{n_{eff}^{2}}=\frac{\cos^{2}\theta }{n_{o}}+\frac{\sin^{2}\theta }{n_{e}} \end{array}$$

Among them, $n_{o}$ is the ordinary light refractive index of the liquid crystal, $n_{e}$ is the extraordinary light refractive index of the liquid crystal, and *θ* is the angle between the direction of light propagation in the liquid crystal and the main direction of the liquid crystal droplets ($n_{e}$ direction). When visible light is incident perpendicularly, the incident light propagates along the main direction of the liquid crystal droplets, and at this time, *θ* is close to 0°. Therefore, according to Equation (6), the effective refractive index of the liquid crystal for the propagating light is $n_{o}$, and the PDLC film appears transparent. As *θ* increases, the effective refractive index of the liquid crystal gradually transitions from $n_{o}$ to $n_{e}$.

**Figure 6:**
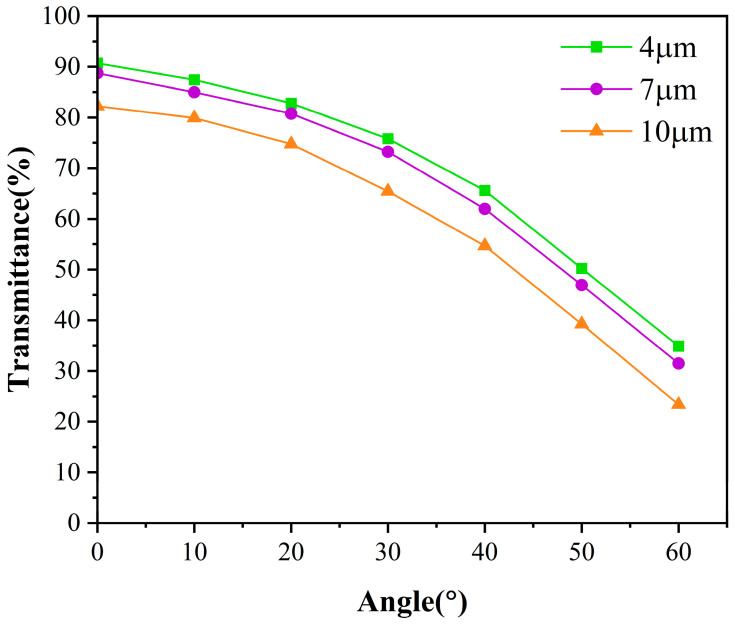
Transmittance of samples with different liquid crystal cell thicknesses under saturation voltage as a function of viewing angle.

Figure 6 shows the variation of transmittance with the viewing angle for PDLC samples with different cell thicknesses under applied saturation voltage. The refractive index of the polymer system is typically defined as $n_{p}$, which is the refractive index of the cured PDLC matrix. The degree of matching between the refractive index of the liquid crystal microdroplets and the polymer matrix directly affects the transmittance (transparent when well matched, scattering when poorly matched) [40]. From Figure 6, it can be seen that at small incident angles, the liquid crystal and polymer exhibit good optical matching, resulting in high transmittance. However, as the incident angle increases, the effective refractive index of the liquid crystal gradually transitions from $n_{o}$ to $n_{e}$, and the difference between the refractive index of the liquid crystal and the refractive index $n_{p}$ of the polymer matrix gradually increases, significantly reducing optical matching and causing a rapid decrease in transmittance.

**Figure 7:**
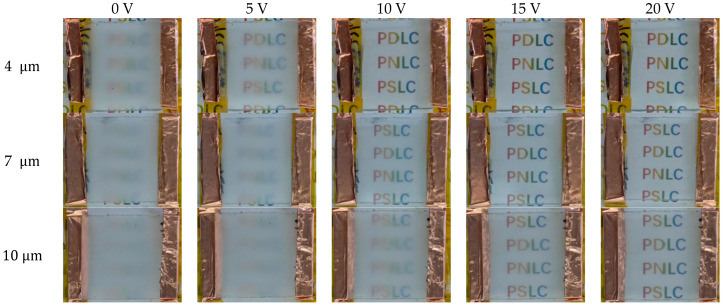
Images of samples with 77 wt% liquid crystal content at different voltages and liquid crystal cell thicknesses.

The thickness requirements for PDLC films vary across different application scenarios. For display applications, low driving voltage is required, and a thinner cell thickness can be selected, whereas for smart window applications, high contrast is required, and a thicker cell thickness can be selected. Figure 7 shows the operating state of a sample with a liquid crystal content of 77 wt% under different cell thicknesses and different voltages.

### 2.3. High-Temperature Electrical and Optical Performance of PDLC

**Figure 8:**
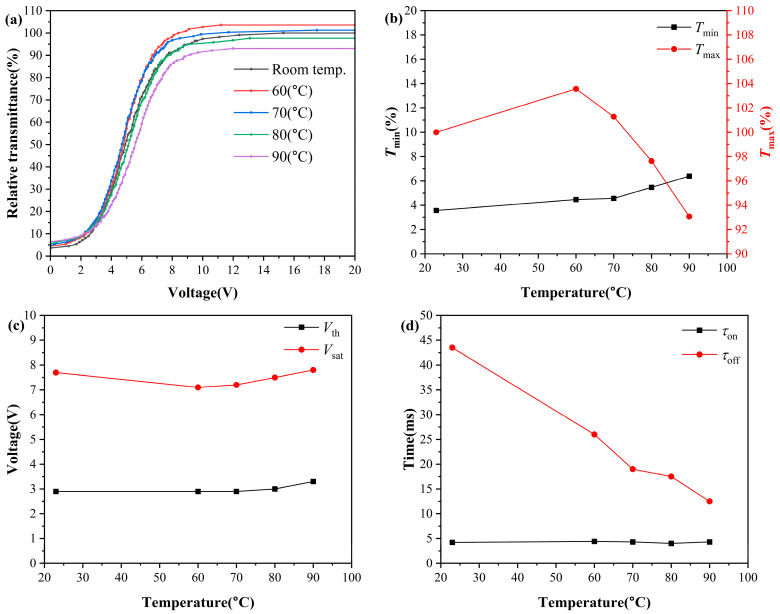
Electro-optical properties of PDLC at different temperatures: (**a**) voltage-normalized transmittance curves; (**b**) maximum and minimum transmittance; (**c**) driving voltage; (**d**) response time.

To evaluate the application potential of PDLC in extreme environments, electro-optical characterizations were carried out on 4-μm-thick PDLC cells with an optimal liquid crystal content of 77 wt% over the temperature range of 23 °C to 90 °C, and the results are displayed in Figure 8. It should be noted that due to systematic differences in the optical path structure of the test system, the absolute transmittance values obtained in this section’s tests exhibit an overall deviation compared to the ambient temperature baseline test results mentioned earlier. Therefore, this paper focuses on analyzing the relative variation of the sample’s electro-optical properties with temperature, and the transmittance is normalized based on the maximum transmittance at room temperature to characterize the high-temperature performance retention of the sample. The test results indicate that this high liquid crystal content PDLC maintains stable electro-optical switching characteristics within the high-temperature range of 60 °C to 90 °C, without exhibiting the severe performance degradation at high temperatures typically observed in conventional PDLC [33].

As the temperature rises, $T_{min}$ continues to increase. This is due to the birefringence Δn of the liquid crystal gradually decreasing with increasing temperature [41], the refractive index difference between the liquid crystal and polymer decreasing, and the scattering efficiency of light passing through the liquid crystal layer decreasing, leading to an increase in off-state leakage. $T_{max}$ exhibits a trend of first increasing and then decreasing with increasing temperature: when heated from room temperature to 60 °C, the viscosity of the liquid crystal decreases with increasing temperature, and the uniformity of liquid crystal molecular orientation under an electric field improves, so $T_{max}$ rises; in the 60 °C~90 °C range, the temperature approaches the clearing point of the liquid crystal, the order of the liquid crystal drops sharply, and the orientation uniformity deteriorates, so $T_{max}$ begins to decrease. As shown in Figure 8b, when the temperature stabilizes at 60 °C, the maximum transmittance of the sample reaches 103.6% of that at room temperature, and when the temperature stabilizes at 90 °C, the sample still maintains a maximum transmittance of 93% at room temperature.

The drive voltage shows a trend of first decreasing and then increasing with temperature rise. From room temperature heating to 60 °C, the interfacial anchoring energy and liquid crystal viscosity decrease with increasing temperature, while the liquid crystal dielectric anisotropy increases [42], and thus the drive voltage continuously decreases; in the 60 °C~90 °C range, the sharp decrease in liquid crystal order leads to a significant reduction in dielectric anisotropy, causing the drive voltage to reverse and rise [21]. As shown in Figure 8c, even at 90 °C high temperature, the $V_{th}$ of PDLC only increased by 0.4 V compared to room temperature, and $V_{sat}$ only increased by 0.1 V, still maintaining excellent low-voltage drive characteristics.

The rise time remains largely unchanged due to the dominant effect of the external electric field, while the decay time continuously shortens with increasing temperature. This is because the rotation viscosity of liquid crystals decreases exponentially with temperature, significantly accelerating the recovery speed of liquid crystal molecular orientation after the electric field is removed [43].

The PDLC films fabricated in this work were found to sustain stable electro-optical switching functionality across the tested temperature range. Even at 90 °C, which approaches the clearing point of the liquid crystal, the films retain 93% of their room-temperature maximum transmittance, with only modest increases of 0.4 V in threshold voltage and 0.1 V in saturation voltage. This level of performance retention at elevated temperatures suggests that the material system may be suitable for smart dimming applications in thermally demanding environments, such as automotive glazing and outdoor architectural windows.

For reference, Table 1 summarizes the $V_{th}$, $V_{sat}$, contrast ratio, and tested temperature range of the PDLC films prepared in this work alongside representative values from several recently reported PDLC systems. As many studies in the literature report data for cell gaps of 10 μm or 20 μm, the values presented for our work in this table correspond to the 10 μm cell thickness, which allows for a more direct contextual comparison. Under these conditions, the PDLC films fabricated in this study exhibit a threshold voltage of 7.3 V, a saturation voltage of 15.3 V, and a contrast ratio of 186.73, while maintaining stable electro-optical switching from room temperature up to 90 °C. These results indicate that the present formulation offers a combination of relatively low driving voltage, wide-temperature operational stability, and considerable optical contrast at a cell gap of 10 μm.

## 3. Materials and Methods

### 3.1. Experimental Materials

The liquid crystal (NLC) QYPDLC-901 was purchased from Qingdao Qiujun Liquid Crystal Material Co., Ltd. (Qingdao, China), with a clearing point ($T_{c}$) of 106 °C and an ordinary refractive index (no) of 1.529, possessing a high birefringence (Δn = 0.264) and positive dielectric anisotropy ($\Delta_{\varepsilon }$ ≈ 10.5) (1 kHz, 20 °C). The epoxy acrylate prepolymer CN131 was provided by Sartomer Ltd. (Guangzhou, China). The monofunctional active diluent o-phenoxyethyl acrylate (OPPEA) and isobornyl acrylate (IBOA) were purchased from Changxing Chemical Materials Co. (Chengdu, China), while the trifunctional crosslinker trimethylolpropane triacrylate (TMPTA) was analytical grade and purchased from Shanghai Aladdin Biochemical Technology Co., Ltd. (Shanghai, China). The photoinitiator 1-hydroxy-cyclohexyl phenyl ketone (PI184) was provided by Shanghai Yinchang Chemical Co., Ltd. (Shanghai, China). The indium tin oxide (ITO) conductive glass (sheet resistance of 10 Ω/□) was supplied by South China Xiangcheng Technology Co., Ltd. (Shenzhen, China). All reagents were used directly without further purification.

### 3.2. The Role of Polymer Components in PDLC Systems

Unlike the phenomenon where PDLC tends to undergo excessive microdroplet coalescence under high liquid crystal content, leading to severe deterioration of electro-optical performance [1], this study prepared a series of PDLC films through the precise molecular design of low-functional acrylic ester pre-polymer matrices, synergistically regulating the polymer-induced phase separation (PIPS) dynamics [13]. Specific polymer components played a crucial role in controlling the microstructure, interfacial properties, and thermal stability of the composite system.

Specifically, the epoxy acrylate prepolymer CN131 serves as the fundamental skeleton of the polymer matrix, not only endowing the film with excellent mechanical strength but also ensuring good adhesion between the film and the ITO substrate. To optimize phase separation dynamics, a monofunctional active diluent, OPPEA, and IBOA were introduced into the system. These diluents effectively reduce the initial viscosity of the prepolymer mixture, promoting uniform dissolution of the high-concentration QYPDLC-901 liquid crystal before ultraviolet light curing. It is worth noting that OPPEA has a high intrinsic refractive index due to its aromatic ring structure. Its addition effectively reduces the refractive index mismatch between the polymer matrix and the liquid crystal in the on-state, thereby maximizing the on-state transmittance of the film and significantly enhancing the contrast [32]. Meanwhile, IBOA, with its rigid bicyclic spatial configuration, significantly improves the glass transition temperature ($T_{g}$) of the polymer network and the dimensional stability of the crosslinked skeleton [23]. This rigid network structure forms the intrinsic foundation for the excellent wide-temperature stability of this PDLC.

Furthermore, the addition of the trimodal monomer TMPTA plays a critical role as a crosslinking agent. During the low-light-intensity, slow-curing process initiated by PI184, TMPTA precisely regulates the crosslinking density of the polymer matrix, providing sufficient steric hindrance to effectively suppress the excessive coalescence of liquid crystal droplets into a bicontinuous phase [50]. This tailored three-dimensional network structure ensures the isolation and uniform dispersion of liquid crystal domains while significantly reducing the anchoring energy at the polymer/liquid crystal interface. The extremely low interfacial friction allows liquid crystal molecules to easily reorganize under a weak applied electric field. In summary, the synergistic interaction among these functional polymer components achieves a synergistic optimization of low driving voltage, high contrast, and extreme temperature tolerance.

**Figure 9:**
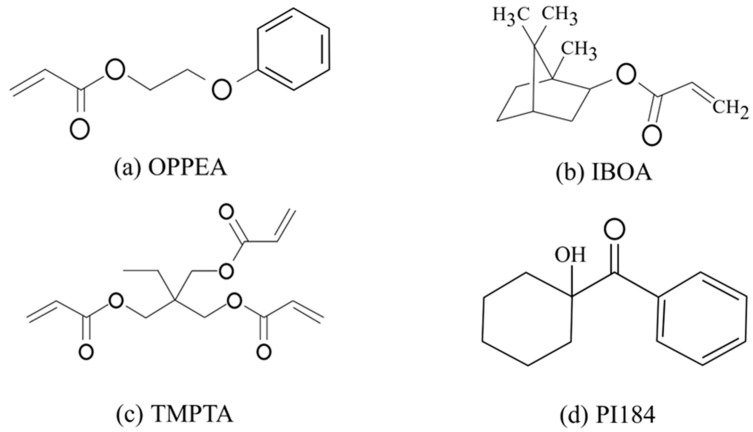
Polymer monomers used in the experiment.

The chemical structures of these monomers, along with the crosslinker TMPTA and photoinitiator PI184, are shown in Figure 9.

### 3.3. Preparation of PDLC Thin Film

This experiment used the phase separation method induced by polymerization to prepare PDLC films. The composition ratio of the samples is shown in Table 2, where QYPDLC-901 liquid crystal and CN131, OPPEA, IBOA, TMPTA, and photoinitiator PI184 were all precisely mixed by weight fraction.

As shown in the table above, this experiment systematically designed a series of PDLC mixtures with different compositional gradients, aiming to deeply investigate the effects of liquid crystal doping concentration and film thickness on the final microstructure and optoelectrical performance of the device. Specifically, the mass fraction of the nematic liquid crystal QYPDLC-901 in the system was precisely set as a gradient variable ranging from 72 wt% to 80 wt%. Meanwhile, the total mass fraction of polymer monomer and initiator correspondingly decreased from 28% to 20%. It should be noted that during the adjustment of the total proportion of the polymer matrix, the relative mass ratio among CN131, OPPEA, IBOA, TMPTA, and PI184 remained constant.

In terms of film-forming parameter control, to isolate the effect of membrane thickness on the concentration effect, the cell thickness of most samples was uniformly controlled at 4 μm as the baseline condition. Additionally, to further reveal the independent mechanisms of the optical path length and the degree of spatial confinement in the polymer network on the electro-optical response characteristics, this study specifically selected the optimized formulation with a QYPDLC-901 content of 77 wt% to prepare a series of films with thicknesses of 4 μm, 7 μm, and 10 μm. Through the above dual-variable (liquid crystal concentration and film thickness) cross-comparison experimental design, a complete PDLC sample library was successfully constructed, providing a reliable material basis for the subsequent systematic characterization of macroscopic photoelectric properties.

**Figure 10:**
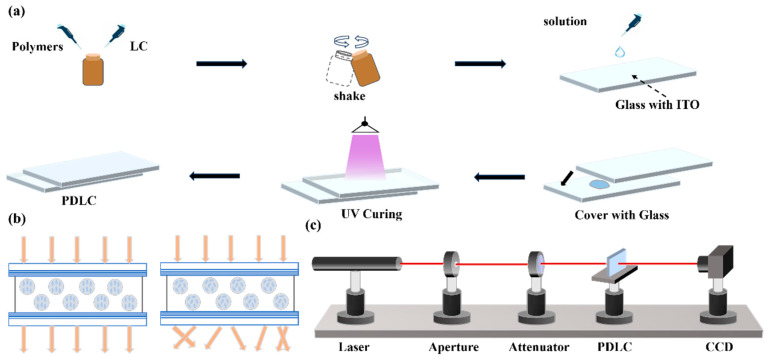
(**a**) Preparation process of PDLC film, (**b**) schematic diagram of PDLC working principle, (**c**) test optical path diagram.

First, mix the polymer components with liquid crystals in a small brown bottle in a proportional manner and place it on a shaker to oscillate until the system becomes uniform. Next, clean a 2 cm × 2 cm ITO conductive glass with anhydrous ethanol, evenly spread 4, 7, 10 μm specifications of spacer pads to prepare three films of different thicknesses; use a pipette to take 4 μL of the above initial mixed solution and add it to the glass surface, slowly cover it with another ITO conductive glass, and then place it under ultraviolet light for 60 s (light intensity 12.8 mW/cm^2^) to complete the preparation of the PDLC film, and perform performance parameter testing at room temperature. The film preparation process is shown in Figure 10a. The preparation conditions are shown in Table 3.

When there is no electric field effect, the liquid crystal droplets are randomly dispersed in the polymer matrix, and the film exhibits a light scattering state; when an external electric field is applied, the liquid crystal droplets align along the direction of the electric field, and the film switches to a transparent state accordingly, this electro-optic response mechanism is shown in Figure 10b.

### 3.4. Characterization Methods

#### 3.4.1. Characterization of Electro-Optical Properties

The electrical optical characteristics of the sample are measured using a self-built optical testing system. As shown in Figure 2c, a He-Ne laser (DH-HN250P) with a wavelength of 632.8 nm is used as the light source, and the sample is placed 32 cm behind the laser. The transmitted light signal is received by a 1 cm × 1 cm photodetector (Dsi300) placed 30 cm behind the sample. To eliminate the influence of ambient background light, the absolute transmittance of the sample is normalized using the transmittance of air as a 100% reference.

The active area of each PDLC cell was defined by the overlapping region of the two ITO-coated glass substrates, which measured approximately 1.5 cm × 1.5 cm. Electrical contact to the ITO electrodes was achieved by attaching copper conductive adhesive tape to the exposed edges of the conductive glass on both sides, which were then connected to the voltage source using alligator clips.

During the test, a square wave AC voltage with a frequency of 1 kHz is applied to the thin film sample. By recording the transmittance–voltage (T-V) curve of the sample, its threshold voltage ($V_{th}$) and saturation voltage ($V_{sat}$) are extracted and analyzed. Additionally, by applying square wave pulse voltage and using a digital oscilloscope (DS1104Z-S) (RIGOL Technologies Co., Ltd., Suzhou, China) to capture the changes in the photodetector signal in real time, the dynamic response time of the liquid crystal molecular reorientation (on-state rise time $\tau_{on}$ and off-state decay time $\tau_{off}$) is precisely measured.

To assess the repeatability of the fabrication and measurement procedures, at least three independently prepared cells were characterized for each experimental condition (liquid crystal content, cell gap, and high temperature). The electro-optical parameters exhibited a high degree of consistency across replicate samples, with the standard deviation of the threshold voltage typically remaining within ±0.1 V. For clarity of presentation, representative curves from a single cell are shown in the figures, while the reported numerical values correspond to the average of the replicate measurements.

#### 3.4.2. Polarized Light Microscope (POM) Characterization

Characterize the microstructure of phase separation within the UV-cured PDLC film using a polarized light microscope (POM). Place the prepared film sample flat on the microscope stage, and under room temperature conditions and orthogonal polarization mode, carefully observe and record the geometric morphology, size distribution, and dispersion uniformity of liquid crystal droplets in the crosslinked polymer network at high liquid crystal content.

#### 3.4.3. Characterization of High-Temperature Electrical Discharge Behavior

**Figure 11:**
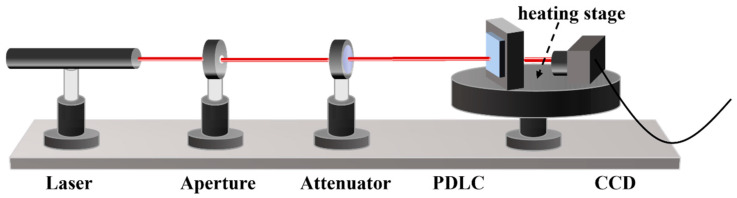
High-temperature experiment optical path diagram.

Room-temperature basic electro-optical performance testing and wide-temperature electro-optical performance testing were completed in two separate photoelectric testing systems. The wide-temperature test requires placing the sample in a temperature-controlled hot stage, where the optical window of the hot stage introduces additional Fresnel reflection and light absorption, as illustrated in Figure 11. Additionally, the optical path alignment has slight deviations. Therefore, the absolute transmittance values measured for the same batch of samples in the wide-temperature testing system exhibit a systematic overall decrease. Moreover, the thicker the sample holder, the longer the optical path, and the greater the amplification of the transmittance deviation caused by the system’s optical path loss. This deviation is a fixed systematic error introduced by the testing system and does not affect the performance change trends and relative change magnitudes of the same sample under different temperatures within the same testing system. The core conclusions of the wide-temperature performance in this paper are based on the relative change patterns within the same testing system.

#### 3.4.4. Contact Angle Characterization

A surface tension meter (DSA-100) (KRÜSS GmbH, Hamburg, Germany) equipped with a high-speed CCD image sensor is used to measure the contact angle of the sample. The instrument can accurately capture the instantaneous morphology of the contact angle between the liquid and the solid surface through a high-resolution optical imaging system and a high-precision liquid dispensing device. First, to prepare the substrate, a 5 μL mixture of polymer and liquid crystal solution is dropped onto a slide and covered with a PET film; after curing for 60 s under ultraviolet light (365 nm, intensity 12.8 mW/cm^2^), the PET film is peeled off to obtain a flat film containing polymer. Subsequently, the contact angle of deionized water with the cured polymer surface at 10 s is measured to evaluate the interfacial anchoring energy of the system.

#### 3.4.5. Representation of Perspective

An optical testing system equipped with a high-precision stepping rotatory stage is used to evaluate the angular characteristics of PDLC films. While maintaining the intensity of the incident laser light source constant, the sample is fixed on the rotatory stage, and the angle between the incident light beam and the normal to the sample surface is continuously varied within the range of 0° to 60°. The relative transmittance data of the sample at different observation angles under the application of a saturated voltage (on-state) are recorded, thereby comprehensively assessing the impact of high liquid crystal content formulations on the multi-directional optical shielding capability and large-angle transparency of the composite film.

### 3.5. Establishment of Photoelectric Performance Characterization Indicators

This study establishes a comprehensive photoelectric performance evaluation system for PDLC devices based on the transmittance-voltage (T-V) characteristic response. In this system, the maximum transmittance ($T_{max}$) and minimum transmittance ($T_{min}$) of the device are used to define characteristic transmittance levels under different states, expressed generally as $T_{x\%}=x\%\cdot \left(T_{max}-T_{min}\right)+T_{min}$. Accordingly, we define the critical electric field strength that triggers the transmittance to reach $T_{10\%}$ as the threshold voltage ($V_{th}$), and the voltage required to drive the device’s transmittance to saturation at $T_{90\%}$ as the saturation voltage ($V_{sat}$). Meanwhile, the optical modulation depth of the device in the switching state is quantitatively measured through contrast ($T_{max}$/$T_{min}$).

In terms of the characterization of dynamically switching properties, the time constant is divided into two stages: turn-on and decay. The turn-on time ($\tau_{on}$) corresponds to the time span during which the transmission rate jumps from $T_{10\%}$ to $T_{90\%}$ under the driving of the electric field; conversely, the turn-off time ($\tau_{off}$) characterizes the period during which the system spontaneously relaxes from $T_{90\%}$ back to $T_{10\%}$ after the driving electric field is removed. In summary, these static thresholds and dynamic time parameters jointly form a solid foundation for evaluating the performance of the device under alternating electric fields.

## 4. Conclusions

This study employs high birefringence liquid crystal QYPDLC-901, successfully fabricating polymer-dispersed liquid crystal (PDLC) films with both low driving voltage and wide-temperature stability through molecular design of a low functionality acrylate system and low-light-intensity polymerization-induced phase separation process. The effects of liquid crystal content, cell thickness, and temperature on the electro-optical properties of the material were systematically investigated. The results show that when the liquid crystal mass fraction is 77%, the device exhibits optimal comprehensive performance, with a threshold voltage as low as 2.9 V, saturation voltage of 7 V, rise time of 4.2 ms, and decay time of 47 ms, achieving the best match of high on-state transmittance and low off-state transmittance. Polarized optical microscopy reveals that this optimal performance coincides with a uniform dispersion of liquid crystal domains. Contact angle measurements further indicate a reduction in solid–liquid interfacial free energy with increasing liquid crystal content, which provides a plausible thermodynamic rationale for the observed low-voltage switching behavior. Increasing the cell thickness can enhance contrast but will raise the driving voltage and extend the decay time. More importantly, the fabricated PDLC films exhibit excellent electro-optical stability within a wide-temperature range of 23 °C to 90 °C, maintaining 93% of the maximum transmittance at room temperature even at 90 °C, with only 0.4 V and 0.1 V increases in threshold and saturation voltages. These modest shifts indicate that the electro-optical response remains relatively stable even at temperatures approaching the liquid crystal clearing point. This work effectively breaks the trade-off between low driving voltage and high-temperature structural stability through molecular design of the polymer matrix and phase separation process regulation, providing important experimental evidence and material foundation for smart dimming devices targeting extreme environmental applications such as automotive smart glass and building curtain walls.

## Figures and Tables

**Table 1:** Comparison of electro-optical performance and temperature stability for representative PDLC systems.

Strategies	$V_{th}$ (V)	$V_{sat}$ (V)	CR	Temperature (°C)	Cell Gap (μm)	Ref.
Our work	7.3	15.3	186.73	RT~90 °C	10	Our work
Fluorine And Cyano-group	≈11	≈18	≈150	−20 °C~90 °C	20	[21]
Functional Nanofibers	18.74	30.38	129.48	RT~90 °C	20	[44]
Thiol Monomer	10.75	21.96	151.91	RT	20	[45]
Curing Temperature Control	≈13	≈21	≈160	−20 °C~90 °C	20	[23]
Rigid Monomer	≈14	≈27	≈50	−20 °C~80 °C	20	[33]
Partitioned Polymerization	≈17.0	25.5	96.6	RT	20	[46]
MoO_2_ doping	13.5	29.0	29.1	RT	8	[47]
Diverse Acrylates	≈9.5	15.8	163	RT	20	[48]
Thiol-Ene Click Reaction	≈13	≈27	≈170	RT	20	[49]

**Table 2:** Material ratio and liquid crystal cell thickness.

Materials Ratio	Polymer: LC Ratio	Cell Gap (μm)
CN131/OPPEA/IBOA/TMPTA/PI184 41/33/20/4/2	28:72	4
	26:74	4
	24:76	4
	23:77	4, 7, 10
	22:78	4
	20:80	4

**Table 3:** Preparation conditions of PDLC films.

UV-A Curing		Magnetic Stirring	
Intensity (mW/cm^2^)	Time (min)	Time (min)	Temperature (°C)
12.8	1	10	60

## Data Availability

The original contributions presented in this study are included in the article. Further inquiries can be directed to the corresponding author.
